# Introduction of IgM testing for the diagnosis of acute Lyme borreliosis: a study of the benefits, limitations and costs

**DOI:** 10.1007/s10096-021-04366-4

**Published:** 2022-01-28

**Authors:** Greg Joyner, Sally Mavin, Rachel Milner, Chin Lim

**Affiliations:** Scottish Lyme Disease and Tick-Borne Infections Reference Laboratory (SLDTRL), Raigmore Hospital, Inverness, IV2 3UJ UK

**Keywords:** Serology, Lyme borreliosis, *Borrelia*, Diagnostics, IgM

## Abstract

Testing for IgM antibodies to *Borrelia burgdorferi* in Scottish patients with suspected Lyme borreliosis was introduced in 2018 to supplement the IgG testing already in situ. Results from 2018 to 2020 were assessed alongside available clinical data to evaluate the utility of IgM testing in serum. An estimated false positive rate of 25.5% was observed with IgM immunoblot vs 80.1% for IgM chemiluminescent immunoassay (CLIA). IgM testing can aid earlier diagnoses if used within a selective two-tier testing protocol: only patients with acute onset of symptoms should be tested for IgM CLIA but confirmation by immunoblot and consideration of clinical picture is necessary.

## Introduction

Serological testing is recommended for all suspected cases of Lyme borreliosis (LB) other than those with clinically identified erythema migrans (EM) rash. Until recently, testing for LB in Scotland utilised a two-tier testing protocol: a screening enzyme-linked immunosorbent assay (ELISA) for the detection of IgG antibodies to *Borrelia burgdorferi* sensu lato (hereafter *B. burgdorferi*) followed by IgG immunoblot [1]. To aid earlier detection of LB and to comply with the National Institute for Clinical Excellence (NICE) guidelines for Lyme disease published in 2018 [2], immunoblot for the detection of IgM antibodies to *B. burgdorferi* was introduced in 2018, followed by the introduction of IgM (and IgG) chemiluminescent immunoassay (CLIA) in 2020.

Methods to detect IgG antibodies to *B. burgdorferi* lack sensitivity during early disease, and their persistence in serum can complicate interpretation [3–8]. Although IgM antibodies are produced earlier than IgG, studies found that IgM tests have suboptimal specificity with high false positive rates due to cross-reactions with other infections and autoantibodies [9–11]. IgM may also persist [12, 13].

The aim of this study was to evaluate the benefits and limitations of both CLIA and immunoblot for the detection of IgM antibodies to *B. burgdorferi* for the laboratory diagnosis of patients with acute LB.

## Methods

Data for serum samples sent from throughout Scotland and tested at the Scottish Lyme Disease and Tick-Borne Infections Reference Laboratory (SLDTRL) for *B. burgdorferi* antibodies from 1 June 2018 to 17 October 2020 were analysed:

(i) Sera from 01/06/2018 to 14/04/2020 were tested by Enzygnost Lyme-link VlsE/IgG ELISA (Siemens) on the DS2 platform (Launch) following the manufacturer’s instructions. Equivocal or positive sera were subsequently tested by Borrelia recomLine IgG and IgM immunoblot (Mikrogen) on the CarL immunoblot platform (Mikrogen) and the results interpreted as per the manufacturer’s instructions. Samples that were IgM immunoblot positive and IgG immunoblot negative or equivocal were identified for further analyses.
(ii) Sera from 15/04/2020 to 17/10/2020 were tested by DiaSorin Borrelia IgG and IgM Quant CLIA on the Liaison XL analyser following discontinuation of the Enzygnost ELISA. Any samples that were positive or equivocal by either assay were tested by IgG and IgM immunoblot as above. Samples that were IgG CLIA negative and IgM CLIA reactive (positive/equivocal), IgM immunoblot positive and IgG immunoblot negative or equivocal were identified for further analyses.

Clinical information from specimen request forms, additional information from questionnaires returned from the referring clinician and any data from subsequent samples were used to allocate individual patients with isolated IgM results into groups based on likelihood of acute LB (Table 1).

**Table 1:** Patient groups and selection criteria for samples with isolated *B. burgdorferi* IgM results

Patient group	Allocation criteria
Probable acute LB	• Clinical history/symptoms: EM rash, tick bite and rash or specific neurological symptoms, i.e. facial palsy • Onset < 10 weeks • Follow-up sample consistent with LB
Possible acute LB	• Clinical history/symptoms: tick bite/exposure and flu-like symptoms • Onset < 10 weeks
Not consistent with acute LB	• Non-specific symptoms and/or not thought to be LB by a clinician (via questionnaires) • Symptoms of late LB, i.e. monoarthritis • Onset > 10 weeks
Insufficient clinical details and data	Insufficient or no clinical details

## Results

**Fig. 1:**
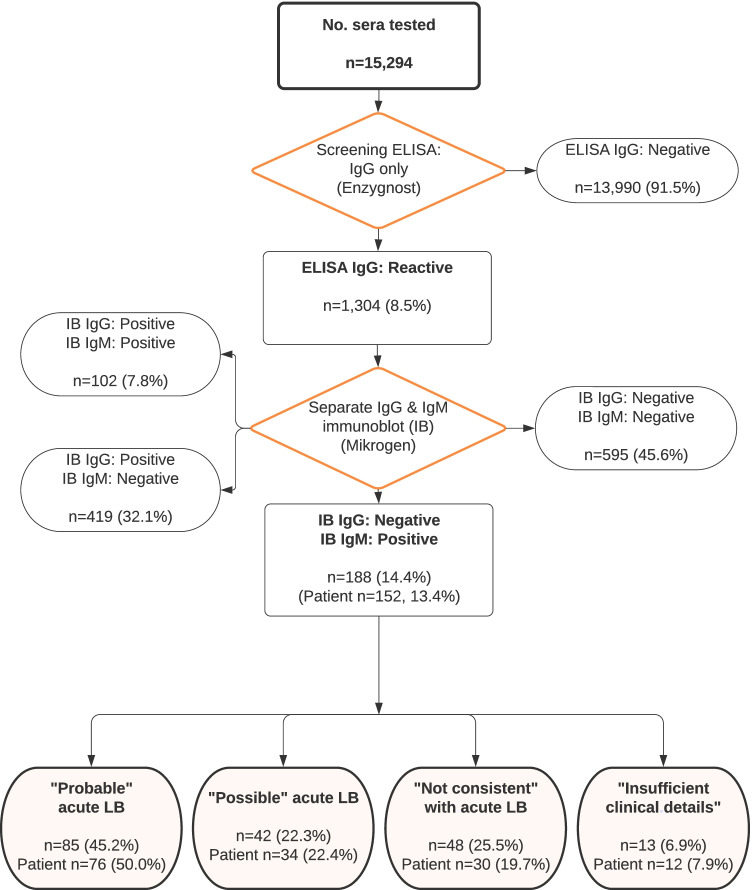
Flow diagram showing the distribution of immunoblot results (1 June 2018 to 14 April 2020) and the allocation of isolated IgM immunoblot patients into the four groups based on the likelihood of acute Lyme borreliosis (LB)

Of the 15,294 sera tested for LB for the period 1 June 2018 to 14 April 2020, 1304 (8.5%) were reactive by IgG ELISA and thus tested by IgG and IgM immunoblot. Of these, 188/1304 (14.4%) were IgM immunoblot positive and IgG negative or equivocal. These 188 sera came from 152 individual patients: 76 (50.0%) were classed as “Probable” acute LB, 34 (22.4%) as “Possible” and 30 (19.7%) as “Not consistent” with acute LB. Twelve (7.9%) patients had insufficient clinical details to assign a presumptive diagnosis (Fig. 1).

**Fig. 2:**
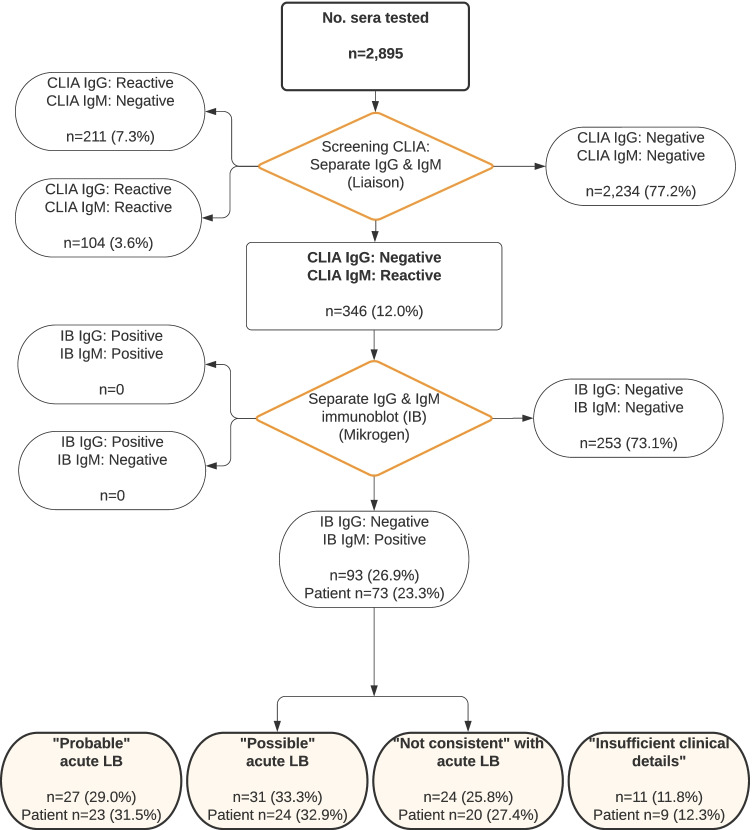
Flow diagram showing the distribution of CLIA results (15 April 2018 to 17 October 2020) and the allocation of positive IgM CLIA confirmed by IgM immunoblot samples and patients into the four groups based on the likelihood of acute Lyme borreliosis (LB)

Of the 2895 sera tested for LB for the period 15 April 2020 to 17 October 2020, 661 (22.8%) were reactive by IgG and/or IgM CLIA and subsequently tested by IgG and IgM immunoblot. Of these, 346 (52.3%) were reactive for IgM CLIA only: the majority of which (73.1%) did not confirm by immunoblot, the remaining 93 (26.9%) sera were positive for IgM immunoblot only. These were from 73 individual patients: 23 (31.5%) patients were classed as “Probable” acute LB, 24 (32.9%) as “Possible” and 20 (27.4%) as “Not consistent” with acute LB. Nine patients (12.3%) had insufficient clinical details to assign a presumptive diagnosis (Fig. 2).

During the first study period (22.5 months), following the introduction of IgM immunoblot, a total of 1304 IgM immunoblots were performed, representing an increase in laboratory consumables costs of £34,556 (£18,430 per year). During the second study period (6 months), following the introduction of IgM CLIA, 2895 specimens were tested by IgM CLIA at an additional cost of £10,595. An additional 346 sera were tested by immunoblot as they had a reactive IgM CLIA, resulting in additional immunoblot costs of £18,338. Thus, the increase in laboratory costs after the introduction of IgM CLIA could be extrapolated to £57,866 per year.

## Discussion

This study has shown that IgM immunoblot is a valuable tool in the laboratory diagnosis of LB, allowing us to detect 110 patients with “Probable” or “Possible” acute LB over the initial 22.5-month study period that may otherwise have been missed with IgG immunoblot alone. Although false positive results were obtained with the IgM immunoblot, the estimated false positive rate of 48/188 (25.5%) was slightly lower than other recent studies [14, 15]. Introduction of IgM immunoblotting led to an increase in test costs of approximately £34,556. However, if more cases of acute LB are detected and treated early, significant cost savings to the health service could result. The increased risk of developing disseminated and late LB, along with the associated manifestations, in untreated patients has been well described [16–18]. Whilst repeat samples are routinely requested in patients with negative serology and recent onset, there is a clear potential for cases to be missed from follow-up. A 2010 study in the Netherlands found that the mean cost of disseminated LB and Lyme-related persisting symptoms was around 5700 Euros per case [19]. Although unlikely, if all 110 of the above patients were missed and progressed to disseminated LB/persisting symptoms, this could equate to 627,000 Euros as well as a huge personal cost.

Introduction of the IgM CLIA produced a much higher false positive rate. Of the 346 sera with an isolated IgM CLIA result, 277 (80.1%) were potentially false positive as they either did not confirm by immunoblot or were assessed as “Not consistent” with acute LB. This highlights that IgM CLIA testing in sera should only be used as part of a robust two-tiered testing protocol with confirmatory testing. The high false positive rate obtained for the IgM CLIA meant that a much higher proportion of samples required immunoblot testing: 22.9% of sera tested by CLIA (April to October 2020) were reactive with IgG and/or IgM CLIA, in contrast with the 8.5% of sera that were reactive with the IgG ELISA in the previous study period. This put extra pressure on the laboratory staff and greatly increased test costs (£28,933). It could be argued that the benefits of IgM CLIA are only marginal and perhaps not cost-efficient; however, 47 patients with probable or possible acute LB were detected, which may otherwise have been missed. Again, although unlikely, if all 47 were missed and progressed to disseminated LB/persisting symptoms, this could equate to 267,900 Euros.

Our results show that there is a significant risk of reporting of inaccurate and misleading results if patients are diagnosed on the basis of an isolated IgM positive result without consideration of clinical details, disease duration and pre-test probability. Prior studies have found that the majority of patients tested for Lyme serology did not meet European or UK clinical case definitions, and recommend that pre-test probability of infection is considered [5, 6, 20]. As a degree of seroprevalence for *B. burgdorferi*–specific antibodies exists in the population, over testing can lead to high false positive rates. The cost of misdiagnosing someone with acute LB based on false positive results, leading to their inappropriate, ineffective or even harmful treatment with antibiotics, and potentially delaying further investigations into the cause of their symptoms, should not be ignored.

The authors concede that the categorisation of patients into “Probable” or “Possible” LB in this study was flawed as there was limited clinical information available. For this reason, assay sensitivity, specificity and positive predictive values could also not be assessed. Many of the samples tested were not accompanied with adequate clinical details such as symptoms and date of onset. The authors accept this may have influenced the study false positive rate and whilst this may indicate that our testing protocols are suboptimal, this is the real-world scenario for a large number of laboratories where demand for Lyme testing is high and illustrates the challenges faced when interpreting results. The authors also recognise that the study results may have been influenced by the actual assays utilised. It is widely recognised that *B. burgdorferi* assays lack inter-assay consensus, particularly IgM assays [21, 22]. Interestingly, one of these studies calculated that a small loss of specificity led to an additional 192,716 immunoblot tests required (4,625,183 Euros), with an additional 6191 IgM false positive results.

Due to the issues outlined above, some countries have, or are considering, stopping the use of IgM testing for LB. However, some cases of early disease may be missed and patient confidence in testing regimes will undoubtedly be affected, which could further fuel the controversy around testing. Some manufacturers claim that IgG antibodies to the VlsE antigen of *B. burgdorferi* can be detected prior to or parallel to the formation of IgM antibodies and are more specific than IgM assays; thus, the use of VlsE/IgG screening assays is sufficient. However, the IgG CLIA used in this study, which contains the VlsE antigen, missed some cases of acute LB. Although complex to implement in laboratories with a high throughput of specimens, perhaps the use of selective testing protocols would be optimal, utilising IgM CLIA only for those patients with an acute onset of specific symptoms within a two-tier testing protocol.

## Data Availability

N/A

## Code availability

N/A
